# Vitamin D Supplementation and Post-Stroke Rehabilitation: A Randomized, Double-Blind, Placebo-Controlled Trial

**DOI:** 10.3390/nu11061295

**Published:** 2019-06-07

**Authors:** Ryo Momosaki, Masahiro Abo, Mitsuyoshi Urashima

**Affiliations:** 1 Department of Rehabilitation Medicine, Teikyo University School of Medicine University Hospital, Mizonokuchi, 5-1-1 Futako, Takatsu-ku, Kawasaki, Kanagawa 213-8507, Japan; 2 Department of Rehabilitation Medicine, The Jikei University School of Medicine, 3-25-8, Nishi-Shimbashi, Minato-Ku, Tokyo 105-8461, Japan; abo@jikei.ac.jp; 3 Division of Molecular Epidemiology, The Jikei University School of Medicine, 3-25-8, Nishi-Shimbashi, Minato-Ku, Tokyo 105-8461, Japan; urashima.mitsu@gmail.com

**Keywords:** stroke, vitamin D supplementation, rehabilitation

## Abstract

Low vitamin D levels are associated with poorer outcomes after stroke. However, it is not clear whether post-stroke vitamin D supplementation can improve these outcomes. In this study, we investigated the effects of vitamin D supplementation on outcomes in hospitalized patients undergoing rehabilitation after acute stroke. A multicenter, randomized, controlled, double-blind, parallel-group trial was conducted from January 2012 through July 2017. One hundred patients admitted to a convalescent rehabilitation ward after having an acute stroke were randomized, and each one received either vitamin D3 (2000 IU/day) or a placebo. The primary outcome was a gain in the Barthel Index scores at week 8. Secondary outcomes were seen in Barthel Index efficiency, hand grip strength, and calf circumference at week 8. Ninety-seven patients completed the study. There were no significant differences in the demographic characteristics between the groups. The mean (±standard deviation) gain in the Barthel Index score was 19.0 ± 14.8 in the supplementation group and 19.5 ± 13.1 in the placebo group (*p* = 0.88). The Barthel Index efficiency was 0.32 ± 0.31 in the supplementation group and 0.28 ± 0.21 in the placebo group (*p* = 0.38). There were no between-group differences in the other secondary outcomes. Our findings suggest that oral vitamin D3 supplementation does not improve rehabilitation outcomes after acute stroke.

## 1. Introduction

Stroke is a major public health problem, and intensive rehabilitation is a key part of post-stroke recovery [1]. Approximately 16 million strokes and 5.7 million stroke-related deaths occur globally each year [2]. Stroke is a major cause of chronic physical impairment and affects a person’s ability to perform activities in daily life. Functional recovery after stroke remains a high priority in health care, but there is limited evidence relating to effective interventions in patients with post-stroke impairment [3].

In previous research, 86%–89% of elderly patients in a rehabilitation facility were found to have vitamin D deficiency [4,5], and several further studies have reported an association between serum vitamin D levels and functional recovery after stroke [6,7,8,9,10,11,12,13]. Vitamin D is a neurosteroid that is thought to play a neuroprotective role because of the widespread distribution of vitamin D receptors on neuronal and glial cells [14,15]. The European Society for Clinical Nutrition and Metabolism recommends regular monitoring of the need for vitamin D supplementation and adequate dietary vitamin D intake in relation to several neurologic diseases [16]. Furthermore, a recent systematic review and additional clinical research have shown that a combination of exercise and vitamin D supplementation improves physical function, including muscle strength [17,18,19]. Therefore, vitamin D supplementation may have additional benefits in terms of rehabilitation after acute stroke.

Two recent small open-label trials performed in an acute care setting in India found that intramuscular injection of high-dose cholecalciferol (600,000 IU) improved scores on various stroke outcome scales and improved survival chance [20,21]. However, no double-blind randomized controlled trial (RCT) has examined the effects of oral vitamin D3 supplementation on the outcomes of rehabilitation following acute stroke.

The aim of this study is to clarify the effects of oral vitamin D supplementation on the outcomes in post-acute stroke patients.

## 2. Materials and Methods

### 2.1. Study Design

Fourteen hospitals in Japan participated in this multicenter, randomized, double-blind, placebo-controlled, parallel-group trial. The study was conducted in accordance with the Declaration of Helsinki, and the protocol was approved by the institutional review boards at both our own institution (approval number 23-185:6646) and the other participating institutions. The trial was registered at the UMIN Clinical Trial Registry (https://center.umin.ac.jp) as UMIN000007002.

### 2.2. Study Population, Eligibility, and Consent

Patients were eligible for inclusion in the study if they were aged 20 years or older, had suffered a confirmed first hemiparetic stroke (infarct, intracerebral hemorrhage, or subarachnoid hemorrhage), had been admitted to a convalescent rehabilitation ward following acute treatment for stroke, and were deemed by the attending physiatrist to require 8 weeks of in-hospital rehabilitation. Exclusion criteria were as follows: A history of calculi in the urinary tract; vitamin D3 or activated vitamin D supplementation before the stroke; osteoporosis; bone fracture; dysphagia or another disorder that would make it difficult to take an oral vitamin D supplement; or an inability to participate in the study in the opinion of the attending physiatrist. In Japan, treatment in a convalescent rehabilitation ward is covered by national health insurance and includes multidisciplinary post-acute rehabilitation [22]. Before enrollment, patients who met the eligibility criteria on admission were required to provide written informed consent after being provided with an explanation of the study’s aims and procedures by the attending physiatrist. In view of previous findings indicating that almost all elderly patients undergoing rehabilitation have vitamin D deficiency [4,5], we did not include the serum 25-hydroxyvitamin D level as an inclusion criterion. We did not specifically include controls for sun exposure. Because we included only patients with disabilities, it was highly unlikely that any of them would go out and receive sun exposure during the course of the study.

### 2.3. Randomization, Blinding, and Intervention

A central computerized procedure was used to randomly assign patients in permutated blocks of four to receive either vitamin D3 or a placebo, both of which were sourced from Zenyaku Kogyo Co., Ltd. (Tokyo, Japan). Each patient was provided with one numbered bottle containing 450 capsules. The patients in the active treatment group received capsules containing vitamin D3 400 IU, and those in the placebo group received capsules containing vehicle only (sesame oil, porcine gelatin, and glycerin). The active and placebo capsules were not identical in appearance. The patients in the active treatment group were asked to take five capsules daily (providing 2000 IU of vitamin D3) at the same time after dinner. The maximum daily vitamin D3 dose is presently set at 2000 IU by the Japanese Ministry of Health, Labor and Welfare. According to the estimated dose-response curve reported by Gallagher et al., a daily vitamin D3 dose of 2000 IU would provide a mean 25-hydroxyvitamin D level of 37 ng/mL after the plateau phase [23]. Therefore, a vitamin D3 dose of 2000 IU/day was used in this study. Blinding of the patients, physiatrists, and the physical therapists who evaluated outcomes was maintained by bottle numbering until the data collection was complete and the database was locked for analysis.

### 2.4. Follow-Up Procedures

On admission, the collaborating physiatrists collected the baseline patient demographic and clinical data, including age, sex, type of stroke (cerebral infarction, cerebral hemorrhage, subarachnoid hemorrhage), comorbidities (hypertension, dyslipidemia, diabetes mellitus, atrial fibrillation), paretic side, and the interval between the acute cerebrovascular event and admission for rehabilitation. On admission and at discharge 8 weeks later, the rehabilitation staff assessed each patient’s Barthel Index score, Brunnstrom stage (arm, hand, and leg on the affected side), hand grip strength (bilaterally), and calf circumference (bilaterally). The Barthel Index was evaluated by rehabilitation staff at the time of discharge. The Barthel Index is a scale that assesses the patient’s ability to perform activities in daily life. Scores range from 0 (totally dependent) to 100 (fully independent) [24]. The Brunnstrom staging system is used to evaluate the degree of voluntary movement that is free from abnormal muscle synergies after recovery from hemiplegia [25]. The Brunnstrom Stage assesses the affected arm, hand, or leg, all of which are rated on a 6-point Likert-type scale. Grip strength was measured using a Jamar analog hand dynamometer with the patient seated, the affected elbow at the side and flexed at 90 degrees, a neutral wrist position, and support beneath the dynamometer. This position, and the calculation of the mean of three trials relating to grip strength have been well-documented as being reliable [26]. Calf circumference is closely related to whole body muscle mass [27] and was measured at its greatest circumference bilaterally in the sitting position with the knee and ankle flexed at 90 degrees with the feet resting on the floor.

### 2.5. Assessment of Outcomes

The primary outcome was a gain in the Barthel Index scores after 8 weeks of rehabilitation and was determined using the following equation: Barthel Index score at 8 weeks − Barthel Index score at admission [28]. The secondary outcomes related to the Barthel Index efficiency; the proportion of patients who showed an improved Brunnstrom stage at 8 weeks, improvement in hand grip strength (from the baseline and the proportion of patients showing improvement) at 8 weeks, and changes in calf circumference (from the baseline and the proportion of patients showing improvement) at 8 weeks. The Barthel Index efficiency was calculated using the following equation: Barthel Index score at discharge—Barthel Index score at admission/length of stay in days [28].

### 2.6. Statistical Analysis

A previous study reported that the minimum clinically important difference for the Barthel Index in patients after stroke was 9 points [29]. We estimated that the minimum clinically important difference for the primary outcome (the mean gain in the Barthel Index) would be 20 points in the placebo group and 29 points in the vitamin D3 group. An equally divided sample of 100 patients was calculated as being sufficient to detect a mean difference in outcome of 9 points, with a standard deviation of 15, a type I error (two-sided) of 5%, and a power of 80%, on the assumption of a 10% loss to follow-up.

Barthel Index efficiency was assessed using an intention-to-treat analysis. Continuous variables were compared using Wilcoxon’s rank-sum test, and categorical variables were compared using the chi-squared test. For the primary outcome, a sub-group analysis according to age and type of stroke was added. *P*-values < 0.05 were considered statistically significant. All reported P-values were two-sided. No adjustments were made for multiple comparisons. All analyses were performed using Stata version 15.0 (StataCorp LP, College Station, TX, USA) at the Division of Molecular Epidemiology, Jikei University School of Medicine.

## 3. Results

**Figure 1:**
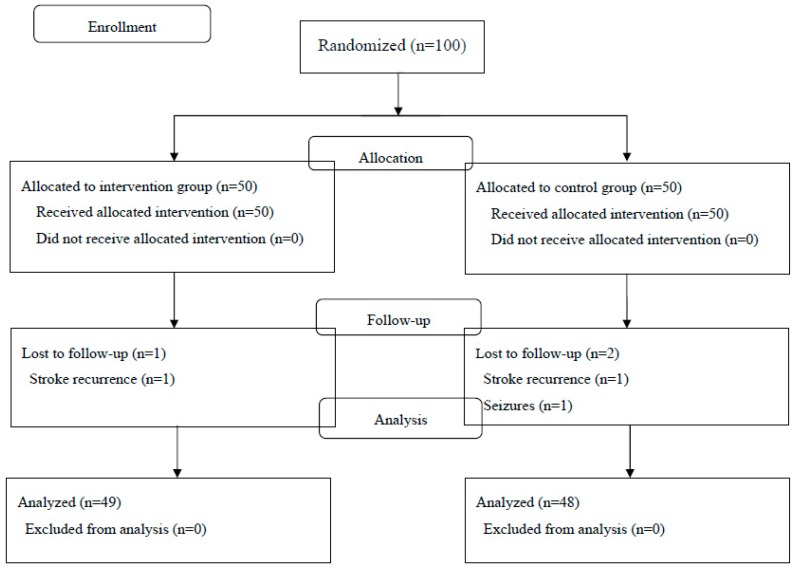
Patient flow diagram.

From January 2012 through July 2017, one hundred patients who met the inclusion criteria agreed to participate in the study and were randomly assigned to receive either vitamin D3 (*n* = 50) or a placebo (*n* = 50). Enrollment can be seen in Figure 1. Three patients were lost to follow-up during the study period because they were transferred to another hospital (one in the vitamin D3 group who developed a cerebral infarction and two in the placebo group because of recurrent stroke (*n* = 1) or the development of seizures (*n* = 1)). Data for 49 patients (98%) in the vitamin D3 group and 48 patients (96%) in the placebo group were available for inclusion in the intention-to-treat analysis. There were no withdrawals because of compliance problems or withdrawal of consent.

Age, sex, type of stroke, comorbidities, paretic side, days since stroke, Barthel Index score, Brunnstrom stage, hand grip strength, and calf circumference were all similar across the two groups at the baseline (Table 1). The mean age of the study population was 67 years old. Of the patients, 70% were male and 59% had suffered a cerebral infarction. The mean interval between the acute cerebrovascular event and admission for rehabilitation was 34 days, and the mean Barthel Index score on admission was 52. The chi-squared and Wilcoxon rank-sum tests did not reveal any significant differences in the baseline characteristics between the two groups.

A comparison of the primary outcome between the two groups is shown in Table 2. There was no significant difference in the mean Barthel Index gain at eight weeks between the Vitamin D3 group and the placebo group (19.0 vs. 19.5, *p* = 0.48). In the subgroup analyses, there was no significant between-group difference in the primary outcome when the patients were stratified by age or type of stroke.

A comparison of the secondary outcomes between the two groups is shown in Table 3. The vitamin D3 group had a higher mean Barthel Index efficiency than the placebo group (0.32 vs. 0.27), but the difference did not reach statistical significance (*p* = 0.46). There was no significant difference in the proportion of patients who showed improvement in the Brunnstrom stage between the groups. Comparison of the right and left sides revealed no significant between-group differences in the change in handgrip strength or calf circumference from the baseline, or in the proportion of patients who showed improvement in hand grip strength or an increase in calf circumference.

## 4. Discussion

In this randomized clinical trial, daily supplementation with 2000 IU of vitamin D3 had no significant beneficial effect on the Barthel Index score in relation to the ability to perform activities in daily life, the Brunnstrom stage of stroke recovery, muscle strength, or calf circumference as an anthropometric parameter in post-acute stroke patients. To our knowledge, this is the first double-blind RCT to examine the effects of oral vitamin D3 supplementation on rehabilitation outcomes in post-acute stroke patients.

In contrast with our present findings, a recent open-label RCT showed that vitamin D deficient patients who received a single intramuscular injection of vitamin D (cholecalciferol 600,000 IU) had an improved Scandinavian stroke scale score at 12 weeks following acute stroke [20]. Another open-label RCT showed that a single intramuscular injection of vitamin D (cholecalciferol 600,000 IU) followed by oral vitamin D (cholecalciferol 60,000 IU) once a month, and elemental calcium, 1 g/day, increased the probability of survival at 24 weeks after acute stroke in patients with vitamin D deficiency [21]. However, in our study, we investigated the effects of short-term daily oral vitamin D supplementation (2000 IU) for eight weeks post-acute stroke. Although a single intramuscular injection of cholecalciferol was shown to increase serum 25-hydroxyvitamin D concentration to significantly higher levels than possible with oral supplementation in the elderly [30], there have been reports of an increased risk of falls and fractures after administration of a single high dose of cholecalciferol [31,32]. Therefore, for safety reasons, we opted to use oral supplementation in our study. There were no withdrawals because of compliance problems in the study, suggesting that oral vitamin D3 supplementation is well tolerated in post-acute stroke patients.

In contrast with the previous RCT treatments of acute stroke, we focused on the post-acute phase. Current concepts of biological recovery after stroke suggest a narrow window of opportunity to encourage brain plasticity and repair [33], so early vitamin D supplementation may make an important contribution to recovery in these patients.

The major limitation of this study was that serum 25-hydroxyvitamin D levels were not measured, so patients without vitamin D deficiency may have been included. When this study began, the Japanese health insurance system did not cover measurement of serum 25-hydroxyvitamin D levels. We abandoned our plan to measure serum 25-hydroxyvitamin D levels, in part because participating facilities refused to carry out the measurements due to the complexity of preserving the collected serum. However, several studies have reported that nearly all elderly patients undergoing rehabilitation have vitamin D deficiency, as mentioned above, so we thought that it would be an acceptable approach to provide vitamin D supplementation without first measuring serum 25-hydroxyvitamin D levels in a clinical setting. There are many countries where it is difficult to routinely measure serum 25-hydroxyvitamin D levels in clinical practice. The results of this study are applicable in such settings.

There were several other limitations. First, the sample size was relatively small. To detect small effects, we needed more participants. Second, only one vitamin D dosage was compared with the placebo. Different outcomes may be achieved using different regimens, including higher doses and longer durations of treatment. Third, our observation period was only eight weeks, so we do not know the long-term effects of low-dose oral vitamin D supplementation or the role of the intermittent use of low-dose vitamin D.

## 5. Conclusions

In this study, oral vitamin D supplementation did not improve rehabilitation outcomes in post-acute stroke patients. Therefore, the use of oral vitamin D supplementation to improve the ability to perform activities in daily life in post-acute stroke patients is not supported.

## Figures and Tables

**Table 1:** Demographic and clinical characteristics of the study population.

	Vitamin D3 (*n* = 49)	Placebo (*n* = 48)
Age, years (SD)	67.6 (11.7)	65.5 (11.7)
Male sex, *n* (%)	34 (69.4)	34 (70.8)
Type of stroke, *n* (%)		
Cerebral infarction	29 (58.3)	28 (59.2)
Cerebral hemorrhage	17 (35.4)	17 (34.7)
Subarachnoid hemorrhage	3 (6.3)	3 (6.3)
Common comorbidities, *n* (%)		
Hypertension	20 (40.8)	17 (35.4)
Dyslipidemia	10 (20.4)	3 (6.3)
Diabetes mellitus	7 (14.3)	4 (8.3)
Atrial fibrillation	2 (4.1)	2 (4.2)
Paretic side, *n* (%)		
Right	25 (51.0)	27 (56.3)
Left	24 (49.0)	21 (43.8)
Days since stroke, mean (SD)	34.2 (16.6)	33.6 (15.6)
Barthel Index, mean (SD)	50.7 (25.9)	53.8 (23.0)
Brunnstrom stage, median (IQR)		
Arm	4 (2–5)	4 (2–5)
Hand	4 (2–5)	4 (2–5)
Leg	3 (2–5)	3 (2–5)
Hand grip strength, kg, mean (SD)		
Right	16.2 (12.9)	17.1 (13.0)
Left	13.8 (14.3)	15.8 (12.1)
Calf circumference, cm, mean (SD)		
Right	32.0 (2.9)	32.3 (3.8)
Left	31.8 (2.8)	32.1 (4.0)

SD, standard deviation; IQR, interquartile range.

**Table 2:** Comparison of the primary outcome (gain in Barthel Index score) between the study groups stratified by age and type of stroke.

	Vitamin D3 (*n* = 49)	Placebo (*n* = 48)	*p*-Value
Barthel Index gain, mean (SD)	19.0 (14.8)	19.5 (13.1)	0.48
Patient age, years			
≥65	16.7 (11.8)	20.2 (12.2)	0.26
<65	24.3 (19.5)	18.8 (14.2)	0.41
Type of stroke			
Ischemic	21.2 (15.7)	17.7 (12.1)	0.75
Non-ischemic	15.9 (13.1)	22.0 (14.3)	0.83

SD, standard deviation.

**Table 3:** Comparison of the secondary outcomes between the groups.

	Vitamin D3 (*n* = 49)	Placebo (*n* = 48)	*p*-Value
Barthel Index efficiency, mean (SD)	0.32 (0.31)	0.27 (0.21)	0.46
Brunnstrom stage improved, *n* (%)			
Arm	21 (42.9)	18 (37.5)	0.59
Hand	17 (34.7)	18 (37.5)	0.77
Leg	20 (40.8)	18 (37.5)	0.74
Hand grip strength			
Right			
Change, kg, mean (SD)	1.4 (3.4)	1.1 (2.8)	0.87
Improved, *n* (%)	27 (55.1)	25 (52.1)	0.77
Left			
Change, kg, mean (SD)	1.2 (3.3)	0.5 (4.1)	0.36
Improved, *n* (%)	20 (40.8)	18 (37.5)	0.74
Calf circumference			
Right			
Change, cm, mean (SD)	−0.1 (1.8)	−0.3 (1.6)	0.99
Improved, *n* (%)	19 (38.8)	20 (41.7)	0.77
Left			
Change, cm, mean (SD)	0.4 (1.6)	−0.2 (2.0)	0.51
Improved, *n* (%)	23 (46.9)	20 (41.7)	0.60

SD, standard deviation.
